# Predictors of mortality and severe illness from *Escherichia coli* sepsis in neonates

**DOI:** 10.1038/s41372-024-02117-9

**Published:** 2024-09-12

**Authors:** Adriana Hoffman, Sriram Satyavolu, Danah Muhanna, Sindhoosha Malay, Thomas Raffay, Anne Windau, Eric M. Ransom, Devashis Mukherjee

**Affiliations:** 1 Rainbow Babies and Children’s Hospital, Case Western Reserve University School of Medicine, Cleveland, OH USA; 2 Case Cardiovascular Research Institute, Cleveland, OH USA; 3 Case Western Reserve University School of Medicine, Cleveland, OH USA; 4 Department of Pathology, University Hospitals Cleveland Medical Center, Cleveland, OH USA; 5 Department of Pathology, Case Western Reserve University School of Medicine, Cleveland, OH USA

**Keywords:** Bacterial infection, Risk factors

## Abstract

Neonatal *Escherichia coli* (*E. coli*) sepsis is increasing. There is limited data on the factors contributing to increased mortality and severity of illness in neonatal *E. coli* sepsis. A retrospective review of neonates (<30 days) admitted to a Level IV NICU in the United States from 2008 to 2022 diagnosed with *E. coli* bloodstream or cerebrospinal fluid infection was conducted. Primary outcome was defined as mortality from or severe illness during *E. coli* infection (defined as a need for inotropic support or metabolic acidosis). *E. coli* neonatal sepsis rate increased from 2008 to 2022 (average of 1.12 per 1000 live births). The primary outcome, which occurred in 57.4% of cases, was independently associated with prematurity, neutropenia, and thrombocytopenia. Ampicillin resistance was not associated with the primary outcome. GA, neutropenia, and thrombocytopenia but not ampicillin resistance, are associated with mortality or severe illness from *E. coli* sepsis.

## Introduction

Neonatal bacterial infections have significant morbidity and mortality, with an estimated annual 6.3 million cases and 230,000 deaths globally [1]. In the United States, the incidence of neonatal bacterial sepsis varies between one and four per 1000 live births (LBs) [2]. Neonatal sepsis is categorized into early-onset (EOS, infants ≤72 h old) and late-onset sepsis (LOS, infants >72 h). EOS classically represents maternal-to-fetal transmission, while most LOS are nosocomial or community-acquired [3]. As antenatal Group B Streptococcus (GBS) screening and intrapartum antibiotic prophylaxis (IAP) have led to significantly decreased GBS EOS, *Escherichia coli* (*E. coli*) is now one of the predominant organisms responsible for EOS [4–7]. Within the National Institute of Child Health and Development (NICHD) Neonatal Research Network (NRN) neonatal intensive care units (NICUs), 36.6% of EOS cases are due to *E. coli* (0.4 per 1000 live births), compared to 30.2% from GBS [4]. The Centers for Disease Control and Prevention (CDC) Active Bacterial Core surveillance data from 2005 to 2014 reported that 24.8% of EOS (0.2 per 1000 live births) in the US were *E. coli* infections [8]. The *E. coli* fraction of EOS cases increases to 58% (NRN) and 44% (CDC) for very low birth weight (VLBW, <1500 g BW) infants. *E. coli* is also the leading Gram-negative organism in LOS (5–12%) in preterm infants in the US [6, 9, 10]. *E. coli* sepsis in neonates is associated with substantial mortality, which is influenced by the gestational age (GA), birth weight (BW), and immune status of the neonate [11–13].

Although the evaluation of suspected EOS has changed over time, the first-line drugs still remain ampicillin and gentamicin [14]. There has been growing concern regarding the emergence of multidrug-resistant *E. coli* strains and reports of increasing resistance to ampicillin and gentamicin [15–17]. The rates of ampicillin and gentamicin resistance in *E. coli* isolates from US NICUs were between 66–80% and 8–17%, respectively [4, 18]. The use of ineffective empiric antibiotics due to resistance results in inadequate infection control and disease progression to severe illness or death

There are inconsistent reports of an association between the risk of death among infants with *E. coli* EOS and ampicillin resistance [19]. It is critical to identify the risk factors associated with mortality and severe illness from *E. coli* sepsis so that antibiotic stewardship programs and aggressive antibiotic regimens for neonates at risk can coexist in the NICU. We determined specific risk factors associated with mortality or severe illness in *E. coli* neonatal sepsis.

## Methods

We conducted a retrospective review of all neonates ≤30 days old with *E. coli* positive blood or cerebrospinal fluid (CSF) culture admitted to Rainbow Babies and Children’s Hospital, a Level IV NICU in Cleveland, Ohio, from 01/01/2008 through 12/31/2022. This study was approved by the Institutional Review Board, and informed consent was waived due to its retrospective nature. Information was collected on infant and maternal clinical and demographic variables. Infants with congenital anomalies and conditions that would predispose to infection, such as immunodeficiency syndromes, were excluded from statistical analysis. Microbiological information included hours of life at culture collection, source (blood vs CSF), time to positivity, and antibiotic resistance profile. Infant clinical characteristics included outcome (discharged home vs death due to *E. coli* infection), complete blood counts (CBC) during sepsis, duration of antibiotic therapy, presence of metabolic acidosis (pH <7.20 or base deficit >10 mEq/L without respiratory acidosis) and need for inotropic medication during the *E. coli* sepsis episode. Maternal characteristics included presence of intra-amniotic infection (IAI, defined as maternal peripartum fever 38–38.9 °C with signs of purulent cervical drainage, maternal leukocytosis, or fetal tachycardia, or isolated maternal fever ≥39 °C), *E. coli* infection in the mother at any time during pregnancy, and maternal intrapartum antibiotic therapy.

We defined our primary outcome as either death or severe illness from *E. coli* infection. Death was attributed to *E. coli* infection if it happened at any point from collection of the blood or CSF culture till the end of antibiotic treatment for that particular episode of infection. Severe illness was defined as the new need for inotropic medication or new development of metabolic acidosis, as described above, during the episode of *E. coli* infection. We specifically included only those infants in our primary outcome who had a new need for inotropic medication and had worsening or new metabolic acidosis at the time of blood or CSF culture at any time during the treatment of the infection as these clinical findings can exist in preterm neonates for reasons other than bacterial sepsis. The time of detection of sepsis, which was used to differentiate between EOS and LOS, was based on the time of collection of blood or CSF culture relative to the time of birth.

### Statistical analysis

Charts that met inclusion and exclusion criteria were checked for completeness and consistency and coded into Rstudio Version 2023.06.1+524. Descriptive data were obtained, and summary statistics were created. These summary tables included the associated *p*-value stratified for the variable of interest. The primary outcome variable was death or severe illness, as defined above. For all analyses, this composite outcome was used for descriptive statistics and logistic regression modeling. Two-way ANOVA was used to evaluate the interaction and main effects of two factors on the dataset. The analysis included multiple comparisons to assess differences between both columns and rows. Bivariate analysis was performed to identify co-linearity between variables of interest. Those variables that were strongly associated (*p* < 0.05) with the outcome variable and relevant to our study were used. Multivariable logistic regression was performed using a backward stepwise method to identify key variables to improve the model’s overall fit to the dataset. The model fitness was assessed using the goodness of fit test to estimate if the model fits the data appropriately. Odds ratios were identified and represented in an odds ratio plot with a 95% confidence interval. This odds ratio plot was used to identify and assess the direction of association between the predictor variables and the outcome variable. *P*-values were reported for each of the given variables in the multivariate logistic regression model. The generalized variance inflation factor (GVIF) was used to identify co-linearity between predictor variables numerically. Those variables with GVIF greater than five were excluded from the model despite these parameters adding significance to the model.

## Results

### Demographics and incidence of *E. coli* sepsis (Table 1)

In the 59,984 live-born infants from 2008 to 2022, there were 68 unique cases of monomicrobial *E. coli* bloodstream or CSF infection in neonates under 30 days old (1.12 cases per 1000 LBs). *E. coli* sepsis incidence per 1000 LBs has steadily increased at our center from 0.35 (7/19,716) in 2008–2012 to 1.03 (21/20 331) in 2013–2017 and to 2.01 (40/19 932) in the last five years (2018–2022) respectively. The incidence of *E. coli* sepsis in VLBW infants was 12.4 per 1000 LBs. There were 38 cases of EOS and 30 cases of LOS. Median GA and BW of infants with *E. coli* sepsis were 31 2/7 wks (27 4/7 – 38 0/7 wks) and 1620 g (970–2835 g) respectively. There were three cases of *E. coli* meningitis; two grew *E. coli* from their blood cultures simultaneously. The median age of diagnosis of *E. coli* sepsis was 31.5 h (1.9–173.8 h).

**Table 1:** Descriptive information on infants diagnosed with *E. coli* bloodstream or cerebrospinal fluid infection from 2008 to 2022.

Demographic/clinical characteristic	
Median GA in weeks (IQR)	31 2/7 (27 4/7 – 38 0/7)
Median BW in grams (IQR)	1620 (970–2835)
Sex, *N* (%)	
Female Male	25 (37%) 43 (63%)
Race, *N* (%)	
Black White Other	37 (54.4%) 28 (41.2%) 3 (4.4%)
Delivery type, *N* (%)	
Vaginal Cesarean section	47 (69.1%) 21 (30.9%)
Inborn Neonates	53/68 (78%)
Maternal IAI	20/68 (29.4%)
Median hours of life at which blood culture was obtained (IQR)	31.5 (1.9–173.8)
Ampicillin resistance, *N* (%)	48/67 (71.6%)
Gentamicin resistance, *N* (%)	6/67 (8.9%)
Mortality	17/68 (25%)
Need for inotropic medication or severe metabolic acidosis	37/68 (54%)
Median days of antibiotics in all infants (IQR)	16 (13–21)
Median days of antibiotics in infants who survived (IQR)	21 (14–21)

*GA* gestational age, *BW* birth weight, *IQR* interquartile range, *IAI* intra-amniotic infection.

### Maternal IAI

Twenty-one infants (30.8%) were born to mothers with a documented IAI. EOS was significantly higher in these infants vs. LOS (*p* < 0.001). Only two *E. coli* LOS cases (without a previous EOS) occurred in infants born to mothers with IAI. There were no differences between rates of mortality, severity of illness, or the primary outcome between infants who were born to mothers with IAI vs those who were not.

### Early and late-onset *E. coli* sepsis

There were 38 EOS and 30 LOS cases during the 15-year study period (Table 3). Males were more likely to have LOS than females, who were more likely to have EOS. The median GA or BW was not different between EOS and LOS infants. Although more VLBW infants were in the EOS group than the LOS group (55% vs 33%, *p* = 0.071), this did not reach statistical significance. There was no difference between ampicillin or gentamicin resistance between EOS and LOS *E. coli* isolates. Mortality was not different between EOS and LOS cases, and neither was a need for inotropic medication or severe metabolic acidosis.

### Primary outcome of mortality or severe illness from *E. coli* sepsis

Thirty-nine (57.4%) infants out of the 68 diagnosed with *E. coli* sepsis met the primary outcome (Table 2). 24/39 (61.5%) of these infants had a BW < 1500 g (VLBW). Out of all VLBW infants diagnosed with *E. coli* sepsis, 77.4% experienced death or severe illness in comparison to 40.5% in infants with BW ≥ 1500 g who were diagnosed with *E. coli* sepsis (OR 5.029, 95% CI 1.729–14.624, *p* = 0.003). The median age (IQR) at which *E. coli* infection was diagnosed for infants with the primary outcome was 37 h (2–168), with 22/39 (56.4%) of these infants having EOS. Twenty-nine of the 39 infants (74.4%) had ampicillin-resistant *E. coli*, which was not statistically different from the degree of ampicillin-resistance in the entire cohort (69.1%), or in the infants who survived to discharge without severe *E. coli* sepsis (62.1%, *p* = 0.28). Apart from GA (and its surrogate marker, BW), the lowest platelet count and the lowest ANC during the episode of sepsis were the only other variables significantly different between those infants who died or had severe illness from *E. coli* sepsis vs those who did not in our univariate analysis (Table 3). In addition, infants who died or had a severe illness were also more likely to be thrombocytopenic or neutropenic during the episode of sepsis. There was no association between the primary outcome and sex, race, ampicillin or gentamicin resistance, mode of delivery, or maternal IAI. None of the three cases of *E. coli* meningitis died or had severe illness. The median antibiotic use duration for *E. coli* sepsis in those infants who survived (with or without severe illness) was 21 days.

**Table 2:** Characteristics of infants who died or had severe illness from episode of *E. coli* sepsis vs. those who survived till discharge without severe illness from *E. coli* sepsis.

Characteristic	Died or had severe illness from *E. coli* sepsis (*N* = 39)	Survived to discharge without severe illness from *E. coli* sepsis (*N* = 29)	*P* value
Median GA in weeks (IQR)	28.5 (25.5, 32.0)	34.5 (30.0, 38.6)	0.002
Median BW in grams (IQR)	1100 (770, 1760)	2410 (1520, 2931)	0.001
Number of VLBW neonates (%)	24 (61.5%)	7 (24%)	0.002
Sex, *N* (%)			
Male	23 (59%)	20 (70%)	0.4
Female	16 (41%)	9 (30%)	
Race, *N* (%)			
White	18 (46%)	10 (34%)	0.5
Black	20 (51%)	17 (59%)	
Other	1 (3%)	2 (7%)	
Delivery type, *N* (%)			0.4
C-section Vaginal	13 (33%) 26 (67%)	7 (24%) 22 (76%)	
Presence of maternal IAI, *N* (%)	11 (28%)	9 (31%)	0.8
Ampicillin resistance^a^, *N* (%)	29/39 (74.4%)	18/28 (64.3%)	0.28
Gentamicin resistance^a^, *N* (%)	4/39 (10.3%)	1/28 (3.6%)	0.3
Lowest ANC, median (IQR)	2.9 (0.9, 5.9)	4.0 (2.4, 6.2)	0.8
Lowest platelet count, median (IQR)	97 (70, 164)	262 (213, 294)	<0.0001
EOS (%)	56%	55%	0.90
Time of collection of blood culture, median hours (IQR)	37 (2,168)	13 (1.5,230)	0.35
Time to positivity of blood culture, median hours (IQR)	9.75 (8.65, 11.38)	9.39 (8.54, 10.70)	0.7
Thrombocytopenic, *N* (%)	25 (64%)	4 (14%)	<0.0001
Neutropenic, *N* (%)	10 (33%)	4 (14%)	0.04

*GA* gestational age, *BW* birth weight, *VLBW* very low birth weight, *IQR* interquartile range, *IAI* intra-amniotic infection, *ANC* absolute neutrophil count, *EOS* early onset sepsis.

^a^1 infant had missing data for antibiotic susceptibility.

**Table 3:** Characteristics of infants with *E. coli* early-onset sepsis (EOS) and late-onset sepsis (LOS).

	EOS (*N* = 38)	LOS (*N* = 30)	*P* value
GA	30.1 (27.4,34.3)	32.4 (26.5, 38.3)	0.40
BW	1380 (1021, 2683)	1775 (791, 2889)	0.50
GA < 37 weeks	29/38 (76%)	19/30 (63.3%)	0.24
VLBW (%)	55%	33%	0.071
Sex			0.011
Female	19/38 (50%)	24/30 (80%)	
Race			0.22
Black White Other	22 16 0	12 15 3	
Delivery type			0.012
C-Section	16/38 (42%)	4/30 (13%)	
Maternal IAI			
Yes	18/38 (49%)	2/30 (7.4%)	<0.001
Ampicillin resistance			
R	28/38 (74%)	19/30 (66%)	0.53
Gentamicin resistance			
R	3/38 (7.9%)	2/30 (6.9%)	>0.9
Mortality	22 (58%)	17 (57%)	0.90
Need for inotropic medication or severe metabolic acidosis	21 (55%)	16 (53%)	0.90

EOS was defined as positive blood or CSF culture within 72 h of birth, and any positive cultures that were obtained after the 72-h period were classified as LOS.

*GA* gestational age, *BW* birth weight, *VLBW* very low birth weight, *IAI* intra-amniotic infection.

**Fig. 1:**
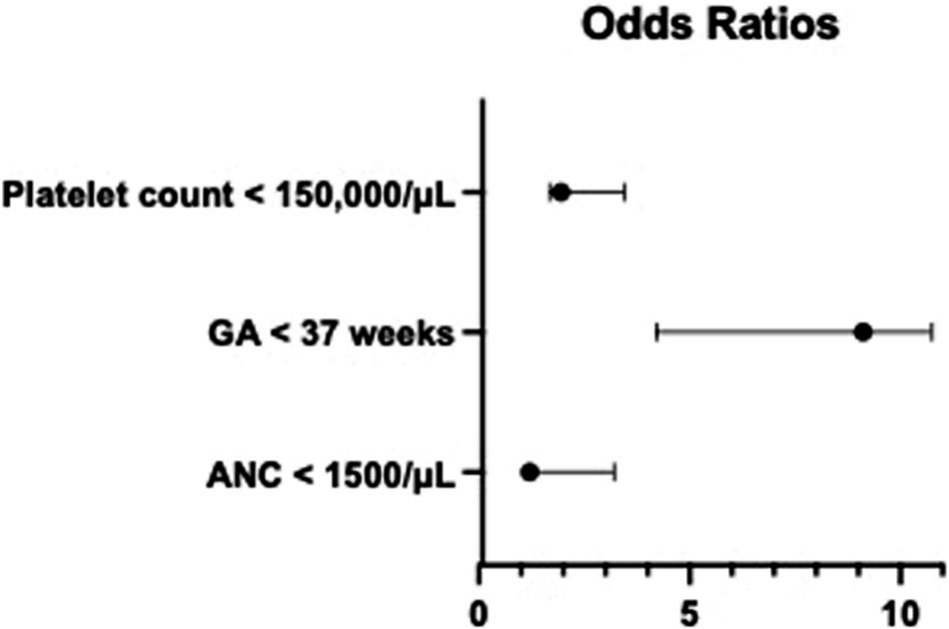
Forest plot of odds ratios of multivariate logistic regression model for factors associated with mortality or severe illness from *E. coli* sepsis in neonates ≤30 days old. OR for platelet count <150,000/µL = 1.95 (1.68–3.46), GA < 37 weeks = 9.11 (4.23–10.75), ANC < 1500/µL = 1.21 (1.13–3.22). GA gestational age.

In our multivariate logistic regression model, the combination of GA < 37 weeks, neutropenia, and thrombocytopenia had four times greater odds of an infant experiencing mortality or severe illness related to *E. coli* sepsis. All three of these variables were individually associated with an increased risk of mortality or severe illness (Fig. 1). GA and BW were co-linear variables, so BW was not included in the multivariate model. Note that sex was used as a variable to improve the overall degrees of freedom, which increased the r-squared statistic.

### Platelet counts and absolute neutrophil counts (ANCs)

Platelet counts and ANCs were available on 64/68 infants with *E. coli* sepsis, out of which 26 infants were thrombocytopenic (<150,000/µL). The lowest platelet count and ANC at any point of time during the entire duration of the sepsis episode was used for analysis. Those who died or had a severe illness were significantly more likely to be thrombocytopenic as compared to those who did not (64% vs. 14%, *p* < 0.0001), with a lower median platelet count during the episode of sepsis (97 (70–164) vs. 262 (213–294), *p* < 0.0001, values in 1000/µL).

Those who died or had a severe illness were also significantly more likely to be neutropenic (<1500/µL) as compared to those who did not (33% vs. 14%, *p* = 0.04). They also had a lower median (IQR) ANC during the episode of sepsis (2.9 (0.9–5.9) vs. 4.0 (2.4–6.2), *p* = 0.8, values in 1000/µL), although this did not reach statistical significance.

### Empiric antibiotic resistance

Antibiotic susceptibility data were available for 67 of the 68 *E. coli* isolates. Ampicillin resistance was 70% overall and similar when including only VLBW infants. Twenty-eight out of 38 (74%) *E. coli* EOS isolates and 19/29 (66%) LOS isolates were ampicillin-resistant. Three out of 38 (8%) *E. coli* EOS cases and 2/29 (7%) LOS cases were gentamicin resistant. Ampicillin and gentamicin resistance did not vary over time. There was no significant difference in our primary outcome between ampicillin-resistant and susceptible cases (29/47, 61.7% vs 10/20, 50.0%, *p* = 0.37). Ampicillin resistance did not differ between VLBW infants and those with BW ≥ 1500 g (22/30, 73% vs. 25/37, 68%, *p* = 0.91), EOS and LOS cases (28/38, 74% vs. 19/29, 66%, *p* = 0.36), infants who met primary outcome vs. those who did not (29/39, 74% vs. 18/28, 64%, *p* = 0.37), and those who died vs. those who survived (12/17, 71% vs 35/50, 70%, *p* = 0.96).

## Discussion

*E. coli* neonatal sepsis accounts for a more significant proportion of cases since the widespread adoption of GBS IAP prophylaxis and subsequent reduction in GBS EOS cases [4, 20]. This is reflected in our study, where the incidence rate of *E. coli* sepsis has increased almost three-fold from 2008–2012 to 2018–2022, despite a similar number of births throughout the entire period. We hypothesize this is predominantly attributable to a global increase in *E. coli* infection in the community, particularly resistant strains [21, 22]. This could potentially lead to increased maternal colonization, leading to more EOS cases, and increased nosocomial transmission, leading to more LOS cases. Although the mortality from *E. coli* disease did not increase in our cohort during this period, the increase in the number of cases is still of concern as the median duration of antibiotic use in infants who survived after *E. coli* sepsis was 21 days. On further chart review, a significant number of the cases where CSF studies could not be obtained due to infant instability were subjected to 21 days of antibiotics for possible meningitis. This is particularly concerning as recent epidemiologic studies have shown that every day of antibiotic exposure in neonates increases mortality and risk of bronchopulmonary dysplasia [23, 24]. In addition, neonatal EOS and LOS may lead to long-term neurodevelopmental impairments in survivors, both in humans and in animal models of disease [25–27]. This is one of the few studies to report the factors associated with the composite outcome of mortality or severe illness from *E. coli* sepsis.

In our univariate analysis, the outcomes of mortality, severe illness, or a combination of either were not associated with sex, maternal race, maternal IAI, maternal intrapartum antibiotic use, delivery type, timing of disease onset, and ampicillin resistance. GA, or BW as a surrogate of GA, thrombocytopenia, and neutropenia, were the only variables significantly different in infants who died or had severe illness, both in the univariate analysis and the multivariate logistic regression model. GA and BW are two variables that have been consistently associated with mortality in neonatal sepsis [28, 29]. Thrombocytopenia is more common in Gram-negative and fungal neonatal sepsis than Gram-positive sepsis. It is associated with increased odds of mortality, especially in Gram-negative sepsis in VLBW neonates [30, 31]. Thrombocytopenia in neonates has been associated with other morbidities, such as IVH and NEC [30, 32]. Thrombocytopenia and neutropenia were both significantly associated with mortality or severe illness in our cohort, both in the univariate analysis and the multivariate logistic regression. Intubation and the need for mechanical ventilation have also often been associated with increased mortality in neonatal sepsis. However, we did not include this variable in our analysis as a majority of these were preterm neonates who were on mechanical ventilation due to their lung disease. We did not have information on the episode-related need for intubation or escalation of respiratory support. There were 15 outborn infants in our cohort, and there were no significant differences in any outcomes when excluding them from analyses. These infants were all transferred from hospitals within our hospital’s health system network.

Antibiotic resistance to *E. coli* strains has been increasing. Although a substantial number of the isolates in our cohort were resistant to ampicillin, our data were similar to recent cohort studies from the NICHD NRN and the CDC, which point to a more than 80% resistance to ampicillin within *E. coli* strains isolated from neonates [4, 8]. Ampicillin resistance did not change over the 15 years we studied. This starkly contrasts with adult studies, where *E. coli* is the most commonly isolated bacterial pathogen in culture-positive community-onset sepsis, but the rates of ampicillin resistance are lower and in the 40–50% range [33, 34]. Based on microbial susceptibility, unit protocols, and drug availability, either cefotaxime, cefepime, or ceftazidime was added to the antibiotic regimens at our center once an isolate was positive for ampicillin-resistant *E. coli*. Although there was no difference in the time to positivity between *E. coli* strains isolated from neonates who met the composite primary outcome vs. those who did not, the prompt switching to a targeted antibiotic could reduce the strength of the finding that ampicillin resistance is not associated with mortality or severity of illness.

Antimicrobial resistance was also similar in VLBW and non-VLBW infants, and we did not find any relationship between ampicillin resistance and death, severe illness, or the composite outcome of either in our study. These data are reassuring, especially in the era of multi-drug resistant *E. coli*, drug shortages, and concern regarding the continued efficacy of ampicillin and gentamicin as first-line empiric antibiotic therapy for EOS in neonates [35]. Neonates with antibiotic-resistant *E. coli* sepsis may also require prolonged hospitalization, which increases the risk of exposure to healthcare-associated infections, including other antibiotic-resistant pathogens. Antimicrobial resistance also leads to longer antibiotic duration and the use of multiple antibiotics, which are independently associated with mortality and morbidity in neonates. Our data point toward the hypothesis that antimicrobial resistance and host-pathogen interaction are possibly exclusive of each other. Mortality and severity of illness are a function of bacterial pathogenicity and host susceptibility. Preterm neonates are susceptible hosts in this context due to their immature developing immune system, which has a dysregulated immune response. Bacterial pathogenicity, which is one of the key drivers of the exaggerated immune response in preterm neonates, leads to endotoxin-mediated tissue damage and hypoxia, metabolic acidosis, cardiorespiratory compromise, organ dysfunction, and ultimately death. Our data lends credibility to the recent AAP consensus statement on the continued use of ampicillin and gentamicin for EOS and warrants caution when attributing causes of sepsis-related death to resistant bacteria [36, 37]. We did find a trend towards increased ampicillin resistance in the neonates who met the primary outcome; however, we cannot comment on whether this would have reached statistical significance with a larger sample size.

A major strength of this study is the extended study period. The incidence of *E. coli* neonatal sepsis is relatively low at any major US NICU. Most studies include NRN, CDC, or VON datasets, which do not account for geographic variations in *E. coli* pathogenicity patterns. We also use a composite outcome of death or severe illness to identify infant and maternal risk factors. As neonatal care becomes better at reducing the outcome of death, there is an increased burden of morbidities in the survivors, and it is critical to include this in studies on sepsis [38, 39]. It is reassuring that ampicillin or gentamicin resistance was not associated with the primary outcome in our study. The rates of ampicillin and gentamicin resistance seen in our population are similar to the rates reported by the recent NRN study, and this rate was similar across EOS and LOS cases as well in VLBW infants who were at the highest risk of death or severe illness [4]. Although this is from a single center, these data suggest that ampicillin and gentamicin remain effective empiric antibiotics for neonatal sepsis.

The main drawback of this study is that this is a single-center experience; thus, the number of *E. coli* cases is insufficient to draw conclusions that can be applied to other centers. We were also limited as to which clinical, demographic, and microbiological factors we could use for our analyses due to the retrospective nature of this study. The completeness of the documentation of these factors in the medical record primarily determined the factors we chose to study. We acknowledge that there are possibly multiple other complex mechanisms and factors that are associated with increased mortality or severity of illness in neonatal *E. coli* sepsis. Maternal factors that have been linked to neonatal death from sepsis are prolonged rupture of membranes, chorioamnionitis, preterm delivery, and cesarean delivery [40]. Neonatal factors that have been consistently shown to be associated with increased odds of mortality are prematurity and low birth weight [41, 42]. Other clinical factors such as respiratory compromise, septic shock, Gram-negative infection, lactic acidosis, and thrombocytopenia have been frequently but not consistently implicated in neonatal sepsis-related mortality [42–45].

The laboratory system at our institution instituted new microbiological techniques with enhanced antimicrobial inactivation to improve the isolation of microorganisms in 2016. This upgrade might partially account for the increased *E. coli* incidence in the last five years. We also do not have follow-up data on the surviving infants, so we cannot speculate on their long-term neurodevelopmental outcomes. However, studies show that hypotension and the need for inotropes are associated with neurodevelopmental impairment (NDI) in preterm infants [23, 46]. We also do not have data on other Gram-negative pathogens, such as *Klebsiella* and *Pseudomonas*, and hence cannot comment on whether there was a similar increase in their incidence during the time period of our study. We also do not have information on the pathogenic strain or phage typing of the *E. coli* species isolated, as this is not routinely reported in our laboratory system.

This study adds to the current wealth of knowledge of *E. coli* sepsis in the neonatal population and the relationship between various host and pathogen factors contributing to poor outcomes.

## Data Availability

Original data is available upon request from the corresponding author.
